# The Pathology of Azoturia (Preliminary Communication)

**Published:** 1900-12

**Authors:** W. R. Brinckerhoff

**Affiliations:** The Sears Laboratory of Pathology


### The Pathology of Azoturia (Preliminary Communication).

W. R. BRINCKERHOFF, S. B.

(*From the Sears Laboratory of Pathology.*)

The material upon which the following description is based was derived from eleven autopsies upon undoubted cases of azoturia occurring in Boston between January 1 and May 1, 1900. The work was undertaken as supplementary to the chemical investigations of Dr. Balch, and is not intended to be a complete description of the pathology of the disease. The liver, spleen, kidney, and psoas muscle only were studied. For control, the organs from three normal horses, three cases of colic, and one of pneumonia were examined.

Gross Anatomy. The organs were examined as soon as possible after the death of the animal, usually within six hours. The following points were observed:

*Liver.* In the acute cases the liver, on section, showed nothing abnormal. When the disease had been of a longer duration the cut surface presented small pale-yellow areas corresponding in distribution to the centres of the lobules.

*Spleen.* In the spleens of two cases there could be felt firm lumps from 1 to 5 cm. in diameter, which on section of the organ were seen to be of a deeper red color than the rest of the spleen substance. These hemorrhagic infarcts have been frequently observed in the spleen in azoturia. In the other cases examined nothing of note was seen in the fresh spleen.

*Kidney.* The examination of the fresh kidney showed nothing but a marked congestion of the organ.

*Psoas Muscle.* In all accounts of azoturia the psoas muscle is spoken of as exhibiting a marked degree of fatty degeneration. In every case examined this muscle on section was notably pale, and bits of it teased out in glacial acetic acid showed many fine and coarse fat drops, both free and within the muscle fibres. The same appearance was noted in a case of pneumonia.

MICROSCOPICAL ANATOMY. *Technique.* All tissues were fixed in Zenker's fluid for twenty-four hours, washed in running water over night, and hardened in 80 per cent. alcohol. The material was then cut into small pieces and passed through two changes of 95 per cent. alcohol, two changes of absolute alcohol, one of chloroform, and one of chloroform saturated with paraffin, remaining in each twenty-four hours. The tissue was then embedded in hard paraffin and cut. Sections from each organ were stained with Unna's methylene-blue and eosin, Mallory's connective tissue stain, and alum hæmatoxylin.

*Liver.* All the livers examined had certain characteristics in common. The capillaries in all were markedly injected. The liver cells contained fine brown granules, and the perivascular spaces were dilated, containing granular material taking the acid stain, presumably precipitated albumin of the serum. This pigment in the liver cells occurs in the livers of normal horses, but not to the extent seen in azoturia. Aside from these slight variations from the normal, two distinct types of lesion were found. In five cases the picture was one of a diffuse process, while in four the lesion was distinctly local in character.

In the first type the centres of the lobules seemed most affected. In the area around the central vein the liver cells were relatively small, distorted, and presented a greater or less degree of vacuolation.

In the second type small areas, bearing no constant relation to the anatomical structures of the organ, were to be seen, in which the liver cells had more or less completely disappeared, the space left being filled with dilated capillaries. Around such areas the nuclei of the liver cells frequently did not stain, the protoplasm being vacuolated and often invaded by leucocytes.

The focal and diffuse lesions were not found associated in any of the cases examined.

*Spleen.* The spleen presented no constant lesion. In seven of the nine examined histologically there was a large amount of brown pigment, in the form of spherules from $\frac{1}{2}\mu$ to $1\mu$ in diameter, enclosed within cells in the pulp. Two spleens showed in the centre of the lymph follicles a circumscribed area composed of epithelioid cells, many of which contained in their protoplasm nuclear fragments. The appearance simulated closely that seen in diphtheria.

*Kidney.* The kidneys exhibited the most constant lesions of any of the organs examined. In every case there was acute parenchymatous degeneration, shown by a more or less extensive degeneration of the convoluted and the collecting tubules. Casts were occasionally found. In six of the ten cases in which the kidney was examined microscopically there was evidence of a chronic interstitial process, consisting in small areas in round cell infiltration, containing atrophied tubules (one case), or in the formation of interstitial tissue around the glomeruli, with occasional bands of connective tissue surrounding atrophied tubules and glomeruli. There was, in several cases, a diffuse increase in the interstitial tissue of the pyramids. In one kidney almost every glomerulus was surrounded by more or less connective tissue.

*Psoas Muscle.* As a matter of routine the psoas muscle was examined histologically in every case. In seven out of ten there was a more or less extensive degeneration of the muscle fibres. The change was selective, attacking some fibres and leaving wide areas of normal structure intact. The affected fibres were unevenly swollen, and contained hyaline areas which were irregularly eroded and often invaded by leucocytes. In a single muscle fibre degenerated portions were seen alternating with apparently normal muscle structure. A dissolution of parts of some fibres had taken place, and the sarcolemma could be traced from one hyaline mass to another, the intervening space containing only a small amount of granular detritus. In some cases there was evidence of repair shown by the presence of young connective tissue cells around the necrotic fibres. In the psoas of the one case of pneumonia examined the same appearance of degeneration was seen, and was there rather more intense than that observed in any of the azoturia series.

SUMMARY. In the eleven cases of azoturia examined changes were found in the liver, spleen, kidney, and psoas muscle. The lesions were:

| | Cases. |
|---|---|
| Fatty degeneration of the liver | 5 |
| Focal necrosis of the liver | 4 |
| Proliferation of the epithelioid cells in the lymph follicles of the spleen | 3 |
| Hemorrhagic infarcts in the spleen | 2 |
| Pigmentation of the spleen | 7 |
| Parenchymatous degeneration of the psoas muscle | 9 |
| Acute parenchymatous degeneration of the kidney | 10 |
| Chronic interstitial nephritis | 8 |

Conclusions. 1. The acute degenerations of the liver and kidney, the proliferation of epithelioid cells in lymph follicles of the spleen, and the focal necroses in the liver are similar to lesions found in man during various of the acute infectious diseases. In the case of the human being such changes are generally regarded as manifestations of a toxæmia.

2. The pigmentation of the spleen indicates nothing more than a destruction of blood corpuscles, and is common to a number of diseases in both man and animals.

3. The degeneration of the psoas muscle is quite analogous to the Zenker degeneration seen in typhoid fever.

4. The chronic interstitial nephritis would indicate either that some toxic substance had acted for a considerable time, or that there had been previous attacks of an acute nature.

We have, then, the picture of a pathological process combining chronic and acute factors. This condition seems most reasonably explained by postulating the presence in the organism of a toxic substance, preferably one capable of causing both acute and chronic changes.

In this study of the lesions found in azoturia, pathological changes have been found which merit further study, and it is my intention at some later date to carry out this investigation in more detail. Experiments upon animals have been begun, but the material at hand from that source is not sufficient to be reported at present, and is also reserved for later publication.

The table formulated by Dr. Balch gives the result of the chemical examinations made by him, the plus (+) mark showing those cases in which the larger amount of lead was found.

The treatment that has been so favorable in these cases of late—the iodide of potassium—is not a digression from the object of this paper, but in my opinion helps to sustain the theory of antiseptic therapeutics.

Works referred to: Finlay Dunn—*Veterinary Medicines*, *Veterinary Record*, *Medical Record*, *American Veterinary Review*. McFarland—*Pathogenic Bacteria*. Hare—*Practical Therapeutics*.

---

Dr. F. F. Hoffman, of Brookville, Pa., recommends in Cæsarean section in sows that the incision in the cornua of the uterus be made perpendicular and not horizontal, as it lessens very much the hemorrhage from the uterine walls.

